# Supplementary material for: High Rates of Sexualized Drug Use or Chemsex among Brazilian Transgender Women and Young Sexual and Gender Minorities
Source: Int J Environ Res Public Health. 2022 Feb 2;19(3):1704. doi: 10.3390/ijerph19031704 (PMC8835457; doi:10.3390/ijerph19031704)
Supplement: Supplementary file 1 [file ijerph-19-01704-s001.zip › ijerph-1546974-supplementary.pdf]

Suppl Table S1. PrEP and PEP information according to gender.

	Total	Gender			p-value
		Cisgender men	Transgender women	Non binary	
	N=3924	n=3553	n=280	n=91	
**PrEP awareness**					**<0.001**
No	185 (4.7)	146 (4.1)	30 (10.7)	9 (9.9)	
Yes	3739 (95.3)	3407 (95.9)	250 (89.3)	82 (90.1)	
**PrEP eligibility**					**0.006**
No	706 (18.0)	630 (17.7)	48 (17.1)	28 (30.8)	
Yes	3218 (82.0)	2923 (82.3)	232 (82.9)	63 (69.2)	
**PrEP use**					**0.011**
Never	2528 (64.4)	2303 (64.8)	165 (58.9)	60 (65.9)	
Current	1139 (29)	1032 (29)	83 (29.6)	24 (26.4)	
Past	257 (6.5)	218 (6.1)	32 (11.4)	7 (7.7)	
**Willingness to use PrEP (N=2528)**					**<0.001**
No	957 (37.9)	818 (35.5)	106 (64.2)	33 (55.0)	
Yes	1571 (62.1)	1485 (64.5)	59 (35.8)	27 (45.0)	
**PrEP modality**					**0.006**
Daily oral PrEP	1048 (92.5)	956 (93)	73 (89)	19 (82.6)	
ED-PrEP	33 (2.9)	31 (3)	0 (0)	2 (8.7)	
Injectable PrEP	52 (4.6)	41 (4)	9 (11)	2 (8.7)	
**Complete adherence (past 30 days) (N=1047)**					0.427
No	358 (34.2)	322 (33.7)	30 (41.1)	6 (31.6)	
Yes	689 (65.8)	633 (66.3)	43 (58.9)	13 (68.4)	
**Complete adherence (past 7 days) (N=1048)**					**0.004**
No	128 (12.2)	111 (11.6)	10 (13.7)	7 (36.8)	
Yes	920 (87.8)	845 (88.4)	63 (86.3)	12 (63.2)	
**PEP use (past 12 months)**					0.857
No	3237 (82.5)	2930 (82.5)	230 (82.1)	77 (84.6)	
Yes	687 (17.5)	623 (17.5)	50 (17.9)	14 (15.4)	

All results are shown in number of respondents in that category followed by percentage in parentheses
